# A Comparison Study of Lymph Node Tuberculosis and Sarcoidosis Involvement to Facilitate Differential Diagnosis and to Establish a Predictive Score for Tuberculosis

**DOI:** 10.3390/pathogens13050398

**Published:** 2024-05-09

**Authors:** Ellen Hoornaert, Halil Yildiz, Lucie Pothen, Julien De Greef, Olivier Gheysens, Alexandra Kozyreff, Diego Castanares-Zapatero, Jean Cyr Yombi

**Affiliations:** 1 Department of Internal Medicine and Infectious Diseases, Cliniques Universitaires Saint Luc, 10 Avenue Hippocrate, 1200 Brussels, Belgium; 2 Department of Nuclear Medicine, Cliniques Universitaires Saint Luc, 10 Avenue Hippocrate, 1200 Brussels, Belgium; 3 Department of Ophthalmology, Cliniques Universitaires Saint Luc, 10 Avenue Hippocrate, 1200 Brussels, Belgium; 4 Department of Intensive Care Medicine, Cliniques Universitaires Saint Luc, 10 Avenue Hippocrate, 1200 Brussels, Belgium

**Keywords:** sarcoidosis, tuberculosis, lymph node, granulomatous diseases, risk factors

## Abstract

**Background:** Tuberculosis (TB) and sarcoidosis are two common granulomatous diseases involving lymph nodes. Differential diagnosis is not always easy because pathogen demonstration in tuberculosis is not always possible and both diseases share clinical, radiological and histological patterns. The aim of our study was to identify factors associated with each diagnosis and set up a predictive score for TB. **Methods:** All cases of lymph node tuberculosis and sarcoidosis were retrospectively reviewed. Demographics, clinical characteristics, laboratory and imaging data, and microbiological and histological results were collected and compared. **Results:** Among 441 patients screened, 192 patients were included in the final analysis. The multivariate analysis showed that weight loss, necrotic granuloma, normal serum lysozyme level and hypergammaglobulinemia were significantly associated with TB. A risk score of TB was built based on these variables and was able to discriminate TB versus sarcoidosis with an AUC of 0.85 (95% CI: 0.79–0.91). Using the Youden’s J statistic, its most discriminant value (−0.36) was associated with a sensitivity of 80% and a specificity of 75%. **Conclusions:** We developed a score based on weight loss, necrotic granuloma, normal serum lysozyme level and hypergammaglobulinemia with an excellent capacity to discriminate TB versus sarcoidosis. This score needs still to be validated in a multicentric prospective study.

## 1. Introduction

Tuberculosis (TB) is a common infectious disease with a high morbidity and mortality worldwide due to *Mycobacterium tuberculosis* (Mtb). In 2021, the disease infected one-third of the world’s population and 10.6 million people had an active disease. Mortality remained worrying, with 1.6 million deaths worldwide (more than 50% in Africa and mostly untreated patients) [1,2]. In 70% of the cases, tuberculosis was a pulmonary disease associated or not with another localization. Of the remaining 30%, half of the cases represented pleural or intra-thoracic lymph node involvement, 5% represented TB meningitis and 8% miliary TB. Therefore, TB has a very important place in the differential diagnosis of extra-pulmonary granulomatous diseases [3].

For the definitive diagnosis of the disease, pathogen demonstration is required but is not always possible. In pulmonary diseases, the diagnosis remains relatively easy and is obtained in 80 to 90% of the cases. The sensitivity of sputum smear microscopy in pulmonary TB is around 60 to 80%, whereas for extra-pulmonary forms, the sensitivity of biopsy sample microscopy is around 25 to 30%. Moreover, the sensitivity of GeneXpert^®^ polymerase chain reaction (PCR) for the identification of TB is higher for pulmonary samplings (98% versus 70%) and with positive sputum smear microscopy as compared to extra-pulmonary samplings and negative microscopy. The role of GeneXpert^®^ PCR has also been established in extra-pulmonary samples. In lymph node samples, sensitivity ranged from 50% to 100%, whereas for cerebrospinal fluid (TB meningitis), the pooled sensitivity and specificity were 62.8% and 98.8%, respectively [4,5,6]. For the diagnosis of joint TB, the sensitivity and specificity of GeneXpert^®^ PCR were 82.65% and 91.00% [4]. Cultures remain also more often negative in extra-pulmonary forms such as in lymph nodes (positivity rate: 38%), bone (positivity rate: 70%), pleural (positivity rate: 25%) and meningeal (positivity rate: 42%) TB, whereas more than 90% of cultures are positive in pulmonary forms [3].

The differential diagnosis with sarcoidosis is therefore challenging, especially in countries with a high burden of TB, because both diseases show several similarities including granulomatous inflammation and overlapping clinical presentations (fever, weight loss, fatigue, respiratory symptoms and ocular involvement). The radiological features of TB are also known to mimic those of sarcoidosis, especially in cases with lymph node involvement [7,8]. There are no specific features sufficient to differentiate both diseases, except a positive culture of Mtb.

The aim of our study was to perform a comparative analysis of demographics, clinical characteristics, laboratory and imaging data, and histological results of patients with lymph node TB and sarcoidosis and to identify characteristics associated with TB. We also aimed to build a predictive score for TB.

## 2. Materials and Methods

### 2.1. Study Design

This retrospective study was performed at a teaching hospital containing 980 beds (Cliniques Universitaires Saint-Luc) in Brussels, Belgium. We identified patients with histologically or microbiologically confirmed sarcoidosis or TB in our institution from January 2011 to June 2022 using our institutional database Medical explorer V8 and Epic electronic health record.

### 2.2. Patient Inclusion and Exclusion Criteria

The inclusion criteria were defined as follows: adult patients ≥18 years with cervical, mediastinal and abdominal lymph nodes who were diagnosed with sarcoidosis or TB, based on the clinician’s judgement. Only patients who underwent 18F-FDG PET/CT were included in this study since one of our objectives was to compare lymph node tuberculosis and sarcoidosis as well as other organ damage.

Patients’ exclusion criteria were defined as patients who did not have a follow up in our institution, patients who did not benefit from an 18F-FDG PET/CT or those whose 18F-FDG PET-CT images or data were not available from the hospital information processing system, patients who did not have lymph node tuberculosis but only pulmonary tuberculosis, and patients with a simultaneous diagnosis of cancer (lymphoma or solid cancer), making it difficult to distinguish between lymph nodes due to sarcoidosis/tuberculosis and those due to cancer.

### 2.3. Ethical Issue

Our institutional ethics committee (Comité d’Ethique Hospitalo-facultaire [CEHF], Clinique Universitaires Saint Luc, Brussels, Belgium) stated that written consent was not needed for the analysis of anonymized data and gave its authorization (n° CEHF 2022/21SEP/348; Belgian registry number: B403). Our study complies with the declaration of Helsinki.

### 2.4. Data Collection

Demographics (age, sex and ethnicity), comorbidities (medication and immunosuppressive state), clinical characteristics (fatigue, fever, arthralgia, dyspnea, cough, erythema nodosum, neurologic symptoms, loss of weight and blurred vision), imaging data (18F-FDG-PET/CT localization of lymph nodes and organ damage (lung, liver, spleen, parotiditis, renal, bone, cutaneous disease…)), microbiological results (culture and GeneXpert), histological results (necrotizing granulomas present or not), and laboratory results at baseline (C-reactive protein (CRP), serum lysozyme, angiotensin-converting enzyme (ACE), QuantiFERON-TB test, lymphocyte count, serum protein electrophoresis, Mantoux (PPD) skin test and calcemia) were obtained retrospectively using our institutional database (Epic electronic health record and Medical explorer V8).

### 2.5. Statistics

Regarding descriptive statistics, continuous variables were expressed as mean and standard deviation, and discrete variables were presented as numbers or percentages. Characteristics between diagnostic groups were compared using the unpaired Student’s t-test, while discrete variables were compared using the Chi-squared test or, where appropriate, the exact Fisher test.

With the aim of discriminating between the diagnosis of TB versus sarcoidosis, a risk model for a binary outcome was constructed based on predictor variables selected by logistic regression. The selected outcome was being diagnosed for TB, and clinically relevant variables were considered predictive factors. Univariate logistic regression was previously performed to identify variables to be included in the multivariate model and to identify the numerical instability and multicollinearity of variables associated with the diagnosis of TB (versus sarcoidosis). Variable selection was performed using a stepwise forward method, with the criterion of a *p* value less than 0.20 for retention in the model. Multivariate analysis was then performed. The results were expressed as odds ratio (OR) with 95% confidence intervals (95% confidence interval [CI]). The risk score for the selected diagnosis was derived by extracting the estimated regression coefficients of each statistically significant variable retained in the final multivariate model. Receiver-operating characteristic (ROC) curves were generated to assess the discriminatory performances of the risk score value in classifying patients into the appropriate diagnosis. Sensitivity and specificity were calculated and the Youden’s J statistic was determined to assess the optimal cut-off value of the risk score.

Statistical tests were two-tailed, and significance was set at the 0.05 probability level. Analyses were conducted using the software program SPSS software (IBM Corp. Released 2011. IBM SPSS Statistics for Windows, version 20.0. Armonk, NY, USA).

## 3. Results

**Figure 1:**
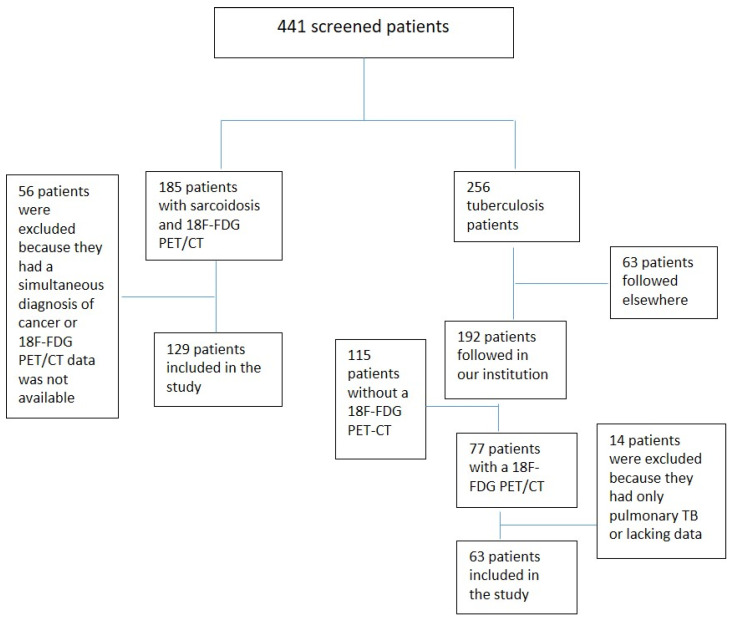
Study flowchart.

A total of 441 patients were screened (256 with tuberculosis and 185 with sarcoidosis) and 192 patients were included in the final analysis: 129 patients with sarcoidosis (67%) and 63 patients with TB (33%) (see the flowchart in Figure 1).

Demographics, comorbidities, and histology, microbiology, lab and imaging results are shown in Table 1.

### 3.1. Demographics

The mean ages of the patients with sarcoidosis and TB were 47 and 44 years, respectively. A total of 46.5% of the sarcoidosis patients were female (*n* = 60) compared to 42.9% in the TB cohort (n = 60).

Concerning the ethnic origins, in the sarcoidosis group, 56.6% (*n* = 73) were Caucasian, while 21.7% (*n* = 28) and 15.5% (*n* = 20) were from the sub-Saharan part of Africa and the north of Africa, respectively. In the TB group, 39.7% (*n* = 25) came from the sub-Saharan part of Africa, 23.8% (*n* = 18) were from the north of Africa and 15.9% (*n* = 10) were Caucasian. There were significantly more non-Caucasian patients in the TB group than in the sarcoidosis group (*p* < 0.05).

### 3.2. Comorbidities

Concerning the comorbidities, 15% of the patients with sarcoidosis had comorbidities, while 43% of the patients had comorbidities in the TB group (see Supplemental File Table S1). There were significantly more HIV patients in the TB group (*n* = 9) compared to the sarcoidosis group (*n* = 1) (*p* < 0.001). No patient was on immunosuppressive treatment at the time of the diagnosis, except one patient on azathioprine for an inflammatory bowel disease (Crohn’s disease), in the sarcoidosis group. In the TB group, 43% of the patients (n = 26) were taking immunosuppressive treatment.

### 3.3. Symptoms

Concerning the symptoms, 25.6% presented with fever in the sarcoidosis group (n = 33) compared to 49.2% (*n* = 31) in the tuberculosis group (*p* = 0.001). A total of 26.4% of the sarcoidosis group patients had arthralgia (*n* = 34) in comparison to 11.1% in the tuberculosis group (*n* = 7) (*p* = 0.016). A total of 9.3% had erythema nodosum in the sarcoidosis group (*n* = 12) compared to 0% in the tuberculosis group (*p* = 0.012). In total, 36% complained of loss of weight in the sarcoidosis group (*n* = 46) and 52% (*n* = 32) did in the tuberculosis group (*p* = 0.036). Blurred vision was present in 32% (*n* = 32) of the sarcoidosis group compared to 5% (*n* = 5) of the tuberculosis group (*p* < 0.001).

### 3.4. Histology

Concerning histological characteristics, 93% of sarcoidosis patients benefited from a lymph node biopsy, whereas only 79.4% of TB patients underwent a lymph node biopsy (other samples: bone and parotid). A total of 93% of the sarcoidosis patients that underwent a biopsy had granulomas, whereas 66% had granulomas in the TB group. A total of 10.4% of the sarcoidosis patients with granulomas were necrotic (*n* = 12). In TB patients, 79% of the patients with granulomas were necrotic (n = 26) (*p* < 0.001).

### 3.5. Labs and Microbiology

Mtb culture and GeneXpert PCR on samplings were negative in all patients with sarcoidosis, while 94% of TB patients had a positive culture (*n* = 59). When GeneXpert PCR Mtb was performed in the TB group, it was positive in 93% of the cases (*n* = 39). It is important to highlight that Mtb culture and GeneXpert PCR in the TB group were performed on all kind of samples and not only on lymph nodes (sputum, bone, peritoneal liquid, EBUS, cerebrospinal fluid, parotid…). Concerning labs results, lysozyme was measured in 87% of sarcoidosis patients (*n* = 111) and was elevated in 54% of the total sarcoidosis group (*n* = 69). In tuberculosis, lysozyme was measured in 30.2% of tuberculosis patients (*n* = 19) and elevated in 12.7% of the total tuberculosis group (*n* = 8) (*p* < 0.001). ACE was elevated in 74% of the total sarcoidosis group (*n* = 95) but was measured in 110 of the 126 patients. In the tuberculosis group, ACE was measured in 30.2% of the patients (*n* = 19) and none of the patients had an elevated ACE level. Polyclonal hypergammaglobulinemia was present in 19.2% of sarcoidosis patients and in 58.2% of the TB patients (*p* < 0.001). The mean CRP level in the sarcoidosis group and the TB group was 24 mg/dL and 39 mg/dL, respectively (*p* = 0.003). Quantiferon was measured in 55 sarcoidosis patients and was positive in 5 of them (9%). In the TB group, Quantiferon was measured in 39% of the patients (*n* = 24) and was positive in 23 out of the 24 patients (*p* < 0.001).

Intradermal reaction (Mantoux (PPD) skin test) was performed in 6.2% of the sarcoidosis patients (*n* = 8) and was negative in all of the patients. Intradermal reaction was performed in 9.5% (*n* = 6) of the TB group and was positive in 4 out of the 6 patients.

### 3.6. Imaging

Concerning the imaging characteristics (analyzed only on 18F-FDG-PET/CT images), symmetrical bilateral mediastinal localization of the lymph nodes was more frequent in the sarcoidosis group compared to the TB group (86.6% versus 23.8%) (*p* < 0.001). Inguinal localization was more frequent in the sarcoidosis group than in the TB group (25.8% versus 7.9%) (*p* = 0.004).

Concerning organ damage, pulmonary involvement was more frequent (61% versus 43%) and was more bilateral (80% versus 50%) in the sarcoidosis group compared to the TB group (*p* = 0.018). The same was observed for splenic involvement (15% versus 4.8%) (*p* = 0.04), uveitis (36% versus 3%) (*p* < 0.001), renal involvement (1.6% versus 0%), cutaneous disease (12% versus 0%) (*p* = 0.005) and arthritis (10.1% versus 0%) (*p* = 0.009). Symptomatic bone involvement was more frequent in the sarcoidosis group (13% versus 1.6%) (*p* = 0.01). On the other hand, asymptomatic bone involvement was more often seen in the TB group (9.5% versus 2.3%) (*p* = 0.027). Adrenal gland involvement was also more often seen in the TB group (4.8% versus 0%) (*p* = 0.012), and so was peritonitis (12.7% versus 0%) (*p* < 0.001).

### 3.7. Univariate and Multivariate Analysis

In univariate logistic regression analysis (Table 2), there was a significant statistical association between TB and the following variables: weight loss, fever, absence of arthralgia, necrotic granuloma, normal serum lysozyme level, elevated CRP level, hypergammaglobulinemia, no symmetrical localization of mediastinal lymph nodes on the 18F-FDG-PET/CT and no bilateral pulmonary involvement on the 18F-FDG-PET/CT (Table 2). Following multivariate analysis (Table 2), only weight loss, necrotic granuloma, normal serum lysozyme level and hypergammaglobulinemia remained significantly associated with TB.

**Figure 2:**
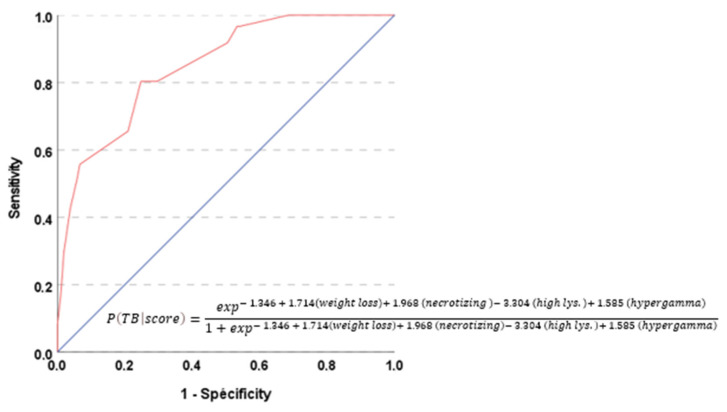
ROC curve assessing the performance of the risk score of tuberculosis. The calculation of the probability of having a diagnosis of tuberculosis based on the score value is represented with the equation of the multivariate logistic regression. A performance analysis revealed an area under the curve (AUC) of 0.85 (95% CI: 0.79–0.91). TB: tuberculosis; necrotizing refers to the variable [*presence of necrotizing granuloma*]; high lys: high value of lysozyme; hypergamma: hypergammaglobulinemia.

With the aim of discriminating between TB versus sarcoidosis, a risk model for a binary outcome was constructed based on the coefficients of the predictor variables selected by logistic regression and is presented in Figure 2. A performance analysis revealed an area under the curve (AUC) of 0.85 (95% CI: 0.79–0.91) as the predictive score capacity to discriminate between the diagnosis of tuberculosis and sarcoidosis. Using the Youden’s J statistic, its most discriminant value was –0.36 and was associated with a sensitivity of 80% and a specificity of 75%.

## 4. Discussion

According to our knowledge, this is the largest comprehensive study comparing lymph node sarcoidosis versus TB with lymph node involvement in 192 cases in a single teaching hospital to help achieve a differential diagnosis and establish a predictive score for tuberculosis (cfr appendix). TB and sarcoidosis are two systemic diseases that share clinical, radiological and histological patterns, and thus, differential diagnosis is quite difficult [7,8].

The most important findings of our study are as follows: in univariate analysis, factors significantly associated with TB were fever, weight loss, absence of arthralgia, necrotic granuloma, normal serum lysozyme level, elevated CRP level, polyclonal hypergammaglobulinemia, no bilateral pulmonary involvement on the 18F-FDG-PET/CT and no bilateral mediastinal lymph node involvement. In multivariate analysis, only weight loss, necrotic granuloma, normal serum lysozyme level and polyclonal hypergammaglobulinemia remained significantly associated with TB.

The literature already showed that both diseases may present with similar constitutional symptoms (fever, weight loss, arthralgia and fatigue), respiratory symptoms, neurological symptoms and ocular manifestations [7,8]. Fever and weight loss were more common in our study in the TB cohort, whereas blurred vision and arthralgia were more prominent in the sarcoidosis cohort. TB patients were also more immunosuppressed (including more patients with untreated HIV disease) and were often originating from high-endemic countries.

Elevated serum angiotensin-converting enzyme (ACE) and lysozyme have been suggested as tools for the diagnosis of sarcoidosis. However, ACE is not elevated in all patients and is not specific since elevated levels are found in granulomatous infections (tuberculosis, leprosy and syphilis), pneumoconiosis (silicosis and berylliosis), deposit metabolic diseases (Gaucher’s disease), endocrine diseases (diabetes mellitus and hyperthyroidism) and liver cirrhosis [9,10,11,12]. A normal value cannot always rule out the condition, even if it is a valuable aid [9].

In our multivariate analysis, normal lysozyme was significantly associated with tuberculosis. Lysozyme can help in the diagnosis of sarcoidosis, as in our cohort, levels were more elevated in the sarcoidosis group (54% versus 12%). Clinicians should keep in mind that a normal value does not exclude sarcoidosis and that levels can be positive in tuberculosis (and even in other infectious diseases such as syphilis) [13]. Lysozyme was found to be much more useful than ACE as a laboratory test to support the diagnosis of ocular sarcoidosis in a study performed by Papasavvas because the number of patients with normal ACE and elevated lysozyme levels were much greater in his study [12].

Belhomme et al. have reported hypergammaglobulinemia in nearly 40% of patients with sarcoidosis (especially with extra-pulmonary involvement) [11]. The sensitivity and specificity of polyclonal antibody activation in sarcoidosis is 70% and 90.4%, respectively [12]. Yet in our study, hypergammaglobulinemia was even higher in the TB group than in the sarcoidosis group and could also help differentiate between both diseases.

A negative Mantoux (PPD) skin test or Quantiferon can also help with differential diagnosis and are considered to suggest sarcoidosis [7]. A negative Mantoux skin test excludes TB, except for in immunosuppressed or extremely ill individuals. Care should be considered also for the interpretation of a negative Quantiferon in elderly, immunocompromised, or chronic and severely diseased patients. Quantiferon can be positive in sarcoidosis patients and be misdiagnosed as TB. In endemic tuberculosis regions, many people have latent TB, detected by Quantiferon [7]. Garneret et al. showed that even a significant proportion of Quantiferon-positive patients (12%) in a large series of sarcoid uveitis patients in a low-endemic country had tuberculosis [14]. Therefore, Quantiferon has been found to be a useful test for the detection of latent TB infection in sarcoidosis patients and one must not make a diagnosis of TB based on a positive Quantiferon alone [7]. In addition, Piotrowski et al. figured out that sarcoidosis activity did not negatively influence the result of the Quantiferon test [15]. Another study revealed that the positivity rate of the Quantiferon test was higher in patients with sarcoidosis than in the general population, which could suggest that *Mycobacterium tuberculosis* might be a factor in sarcoidosis etiology [16]. In our cohort, all sarcoidosis patients tested had a negative PPD test but five had a positive Quantiferon test (one patient of Indian ethnicity and four African patients).

Imaging, and in particular 18F-FDG-PET/CT, can also be a helpful tool for differential diagnosis. In our study, bilateral, symmetrical lymph node enlargement was usually seen in sarcoidosis on the 18F-FDG-PET/CT, whereas unilateral pulmonary disease was more frequent in TB. CT scans of the mediastinum show significant differences in the distribution of lymph nodes in TB and sarcoidosis. Possible explanations for these differences include the route of lymphatic drainage of pulmonary TB [17]. Lung patterns of sarcoidosis and tuberculosis on a CT chest scan can be similar. One of the ways of dissemination of TB is the lymphangitic pathway, which results in micro-nodules with peri-lymphatic distribution and unusual CT features mimicking sarcoidosis, with a low culture of M. tuberculosis [18,19]. Additionally, fibrosis and miliary distribution, although common in TB, may be seen in both diseases [7]. So, traditional imaging has a lot of limitations. 18-FDG-PET/CT at diagnosis is positive in 97.6% of patients with confirmed lymph node TB (it detects more lesions than CT) and discovers unknown lesions in 53.7% of cases [3]. It is also important to assess the activity of the lesions, guide biopsy and determine disease extent [1,3,20,21,22]. In Sarda-Mantal et al.’s study, SUV_max_ on 18-FDG-PET was the best criterion to discriminate between healing and residual disease at the end of treatment and was confirmed in Harkirat, Vorster and Ankrah’s studies [1,3,18,22]. In the future, new, more specific radiotracers, like positron emitter-labeled anti-tuberculous drug molecules, may help to differentiate TB from sarcoidosis [1].

GeneXpert Mtb PCR has good specificity (even if false positives can be obtained with PCR Mtb due to contamination) but its sensitivity is not always optimal (ranging from 50 to 100%) in non-pulmonary specimens, as already revealed in a lot of studies and particularly in Dhooria et al.’s study (49.1%) [4,5,6]. In our study, PCR was positive in 93% of the tuberculosis cases but we included pulmonary samples (sputum; bronchoalveolar lavage), peritoneal fluid, cerebrospinal fluid, bone samples and not exclusively lymph node samples. Culture positivity is the most helpful test because it confirms with certitude the diagnosis of tuberculosis [23], but it has many limitations. It takes a lot of time (3 to 6 weeks), which can be problematic when the patient is unstable, and is not always positive (culture positivity rate in lymph nodes: 38%) [3]. In our study, 94% of the cultures were positive, again in all types of samples as already mentioned.

Both diseases show granulomatous inflammation in histopathology, but necrosis is more often seen in tuberculosis. In the literature, fibrinoid necrosis can occur in up to 30% of cases of sarcoidosis (in our cohort, this number was 10%), so the presence of necrosis in samples is not a reliable finding for a definitive diagnosis [7,24].

Other markers not evaluated in our study, such as high calcium and low iron levels in lymph tissue or an elevated neutrophil/lymphocyte ratio (NLR), may be suggestive of tuberculosis disease compared with sarcoidosis [8,25].

With this study, we concluded that no test or clinical characteristic or sign could confirm TB nor sarcoidosis with certitude, except for the identification of the pathogen through culture. Our study is different from the others because based on the factors’ univariate and multivariate analysis, we tried to establish a predictive score of TB in terms of granulomatous lymph nodes in order to not lose time waiting for the culture results. Our score (cfr appendix) showed that it has the capacity to discriminate between the diagnosis of tuberculosis and sarcoidosis, with an area under the curve (AUC) of 0.85 (95% CI: 0.79–0.91). Using the Youden’s J statistic, its most discriminant value was –0.36 and was associated with a sensitivity of 80% and a specificity of 75%. This is the first time that such a score is described.

Our study has several limitations: This is a single center and retrospective study. Additionally, a larger number of sarcoidosis patients were included compared to tuberculosis patients because Belgium (like other European countries) has a lower incidence of tuberculosis compared to other countries of the world (for example, African countries or India). Finally, this study is based only on the differential diagnosis of sarcoidosis and tuberculosis and did not include malignancies.

## 5. Conclusions

The diagnosis of granulomatous inflammation in lymph node biopsies is common but a differential diagnosis remains challenging. We present a comprehensive study investigating a differential diagnosis between both diseases in which all cases were evaluated clinically, radiologically, biologically and pathologically. We found in multivariate analysis that weight loss, necrotic granuloma, non-elevated lysozyme and hypergammaglobulinemia remained significantly associated with TB. A risk score of TB was built based on these variables. Our score showed that it has the capacity to discriminate between the diagnosis of tuberculosis and sarcoidosis, with an area under the curve (AUC) of 0.85 (95% CI: 0.79–0.91). This score still needs to be validated in a prospective study.

## Figures and Tables

**Table 1:** Demographics, comorbidities, and histology, microbiology, lab and imaging results.

Variables	Total (N = 192) Total Effective (%) or Mean (SD)	Sarcoidosis (N = 129) Total Effective (%) or Mean (SD)	Tuberculosis (N = 63) Total Effective (%) or Mean (SD)	*p*-Value
Gender (women)	87 (46)	60 (46.5)	27 (42.9)	0.63
Ethnic origin				<0.001
African (Sub-Sahara and south of Africa)	53 (27.6)	28 (21.7)	25 (39.7)	
American	5 (2.6)	2 (1.6)	3 (4.8)	
Asiatic	12 (6.3)	5 (3.9)	7 (11.1)	
Caucasian	83 (43.1)	73 (56.6)	10 (15.9)	
North of Africa	35 (18.2)	20 (15.5)	15 (23.8)	
Age, mean (SD)	46.4 (16.1)	47.7 (15)	43.8 (18)	0.11
HIV	10 (5)	1 (0.8)	9 (14.3)	<0.001
Symptoms				
Fatigue	123 (64)	85 (66)	38 (61)	0.63
Fever	64 (33)	33 (25.6)	31 (49.2)	0.001
Arthralgia	41 (21)	34 (26.4)	7 (11.1)	0.016
Dyspnea	55 (29)	40 (31)	15 (23.8)	0.3
Cough	51 (27)	32 (25)	19 (30)	0.43
Erythema nodosum	12 (6.3)	12 (9.3)	0 (0)	0.012
Neurologic symptoms	25 (13)	19 (15)	6 (9.5)	0.31
Weight loss	78 (41)	46 (36)	32 (52)	0.036
Blurred vision	44 (23)	41 (32)	3 (5)	<0.001
SICCA	9 (4.7)	9 (7)	/	/
Organ damage *				
Parotideal glands	19 (9.9)	16 (12)	3 (4.8)	0.1
Liver	34 (18)	25 (19)	9 (14)	0.38
Spleen	22 (11.5)	19 (15)	3 (4.8)	0.04
Uveitis	48 (25)	46 (36)	2 (3)	<0.001
Asymptomatic bone localization	9 (4.7)	3 (2.3)	6 (9.5)	0.027
Symptomatic bone localization	18 (9.4)	17 (13)	1 (1.6)	0.01
Adrenal gland	3 (1.6)	0 (0)	3 (4.8)	0.012
Cutaneous disease	15 (7.8)	15 (12)	0 (0)	0.005
Kidney	7 (3.6)	2 (1.6)	0 (0)	
Bilateral pulmonary disease	73 (38)	57 (80)	16 (50)	0.018
Cardiac	13 (7)	9 (7)	4 (6.3)	0.87
Muscle	7 (3.6)	5 (4)	2 (3.2)	0.81
Central nervous system	21 (11)	17 (13.2)	4 (6.4)	0.16
Hypophysitis	4 (2.1)	4 (3.1)	0 (0)	0.16
Meningitis	16 (8.3)	12 (9.3)	4 (6.3)	0.49
Medullary	2 (1)	1 (0.8)	1 (1.6)	0.6
Polyneuropathy	4 (2.1)	4 (3.1)	0 (0)	0.16
Joint	13 (6.8)	13 (10.1)	0 (0)	0.009
Histology and laboratory results				
Biopsy performed	170 (88.5)	120 (93)	50 (79.4)	0.005
Presence of granuloma	145 (76)	112 (86)	33 (52)	<0.001
Necrotic granuloma	38 (20)	12 (9)	26 (41)	<0.001
Culture BK positive	59 (45)	0 (0)	59 (94)	<0.001
PCR BK positive	39 (20.3)	0 (0)	39 (93)	<0.001
Bronchoalveolar lavage	39 (20.3)	18 (14)	21 (33)	0.006
Elevated CD4/CD8	9 (4.7)	9 (7)	0 (0)	0.032
Hypercalcemia	10 (5.2)	7 (5.5)	3 (4.8)	0.84
Lysozyme measured	130 (68)	111 (87)	19 (30.2)	<0.001
Elevated lysozyme	77 (40.1)	69 (54)	8 (12.7)	<0.001
ACE measured	130 (68)	110 (86)	19 (30.2)	<0.001
Elevated ACE	33 (17.2)	95 (74)	0 (0)	<0.001
QUANTIFERON (MEASURED)	79 (41)	55 (43)	24 (39)	0.61
QUANTIFERON positive	28 (15)	5 (3.9)	23 (36.5)	<0.001
PPD measured	14 (7.3)	8 (6.2)	6 (9.5)	0.41
PPD positive	4 (2.1)	0 (0)	4 (6.3)	0.011
Hypergammaglobulinemia	59 (31)	23 (19.2)	36 (58.1)	<0.001
CD4 (total) N = 78; mean (SD)	456 (306)	541 (305)	376 (294)	0.018
Ly count at diagnosis; mean (SD)	1.38 (0.64)	1.403 (0.62)	1.33 (0.68)	0.49
CRP; mean (SD)	29 (50)	24 (47)	39 (55)	0.003
Death linked to sarcoidosis or TBC	4 (2.1)	3 (2.3)	1 (1.6)	0.74
Death	10 (5.2)	6 (4.6)	4 (6.3)	0.62
18F-FDG PET/CT				
Lymph node localization	192 (100)	129 (100)	63 (100)	/
Sus diaphragmatic localization	86 (44.8)	56 (43)	30 (47.6)	0.43
Sus and sub-diaphragmatic localization	100 (52.1)	69 (53.5)	31 (49)	
Sub-diaphragmatic localization	5 (2.6)	4 (3.1)	1 (1.6)	
Bilateral mediastinal localization	125 (65.1)	110 (86.6)	15 (23.8)	<0.001
Axillary	36 (19)	25 (19.4)	11 (17.5)	0.75
Cervical and sus-clavicular	85 (44.3)	52 (40.3)	33 (52.4)	0.11
Inguinal	38 (20)	33 (25.8)	5 (7.9)	0.004
Sub-diaphragmatic (hepatic and retroperitoneal)	104 (54.5)	72 (56.3)	32 (50.8)	0.48

Ly: lymphocyte, ACE: angiotensin-converting enzyme, CRP: C-reactive protein, PCR: polymerase chain reaction, BK: Koch’s bacillus, and SICCA: dry eye and or mouth. SD: standard deviation. 18-FDG PET/CT: fluorine-18 fluorodeoxyglucose positron emission tomography/computed tomography. PPD: purified protein derivative. * organ damage in sarcoidosis corresponded to the criteria defined as highly possible, at least possible or possible in the WASOG assessment instrument.

**Table 2:** Univariate logistic regression analysis and multivariable logistic regression analysis.

Variables	Univariate			Multivariate		
	OR	95% CI	*p*-Value	Adjusted OR	95% CI	*p*-Value
Weight loss	1.92	1.04–3.55	0.037	5.55	1.55–19.86	0.008
Fever	2.81	1.49–5.30	0.001			
Absence of joint pain	0.34	0.14–0.84	0.019			
Necrotic granuloma	5.97	2.73–13.03	<0.001	5.55	1.69–30.23	0.007
Normal serum lysozyme level	0.122	0.05–0.27	<0.001	0.037	0.007–0.184	<0.001
Elevated CRP level	1.006	1.00–1.01	0.061			
Hypergammaglobulinemia	5.839	2.96–11.51	0	4.882	1.307–18.235	0.018
No bilateral pulmonary involvement on the 18F-FDG-PET/CT	0.246	0.09–0.60	0.02			
No symmetrical localization of mediastinal lymph nodes on the 18F-FDG-PET/CT	0.048	0.02–0.15	<0.001			

## Data Availability

All of the data presented in this study are available on request from the corresponding author. The data are not publicly available due to privacy restrictions.

## Supplementary Materials

The following supporting information can be downloaded at: https://www.mdpi.com/article/10.3390/pathogens13050398/s1, Table S1: Comorbidities in sarcoidosis and TB patients.
