# Supplementary material for: A Comparison Study of Lymph Node Tuberculosis and Sarcoidosis Involvement to Facilitate Differential Diagnosis and to Establish a Predictive Score for Tuberculosis
Source: Pathogens. 2024 May 9;13(5):398. doi: 10.3390/pathogens13050398 (PMC11124455; doi:10.3390/pathogens13050398)
Supplement: Supplementary file 1 [file pathogens-13-00398-s001.zip › pathogens-2996615-supplementary.pdf]

Table S1: Comorbidities in sarcoidosis and TB patients

Comorbidities in sarcoidosis patients	Immunosuppressive treatment	Comorbidities in TBC patients	Immunosuppressive treatment
HIV (n=1)	/	HIV (n=9)	
inflammatory bowel diseases (IBD) (n=4)	Aziathropine (n=1)	Hepatitis C virus (n=2)	Interferon (n=1)
Fabry disease (n=1)	/	active HBV infection (n=1)	
Psoriasis (n=1)	/	mixed connectivitis (Sharp disease) (n=1)	corticosteroids
Diabetes type I (n=2)	/	Psoriasis (n=2)	Humira (n=2)
haemolytic anaemia and atrophic polychondritis (n=1)	/	acute lymphoblastic leukemia (LLA) (n=1)	Chemotherapy (n=1)
myopathy of unknown origin (n=1)	/	Spondylarthritis (n=1)	Remicade (n=1)
lymphoma (n=2)	/	Sarcoidosis (n=1)	Corticosteroids (n=1)
G6PD deficiency (n= 1)	/	Renal graft (n=2)	Tacrolimus, corticosteroids
breast cancer (n=1)	/	IBD (n=1)	Anti-TNF (n=1)
past medical history of tuberculosis	/	LMA (n=1)	Decitabine (n=1)
		Rheumatoid Polyarthritis (n=1)	Arava (n=1)
		chronic renal disease (n=2)	Dialysis (n=1)
		Histiocytosis (n=1)	Vinblastine (n=1)
